# The time course of changes induced by resistance training and detraining on muscular and physical function in older adults

**DOI:** 10.1186/s11556-015-0153-8

**Published:** 2015-10-29

**Authors:** Carla Coetsee, Elmarie Terblanche

**Affiliations:** Department of Sport Science, Faculty of Education, Stellenbosch University, Private Bag X1, Matieland, 7601 South Africa

**Keywords:** Muscle strength, Functional performance, Exercise capacity, Older adults

## Abstract

**Background:**

It is generally recognised that the physical functioning of older adults is enhanced with resistance exercise. The aim of this study was to investigate the time course of changes in upper and lower body muscle strength and physical function in older individuals following a 16 week resistance training (RT) programme and a similar duration detraining (DET) period.

**Methods:**

Forty-one inactive individuals (55 to 75 years) were randomly allocated in a RT group (*n* = 22; three sessions per week) and a control (CON) group (*n* = 19). Muscle strength was assessed with 10RM leg and bench press tests, while the Timed-Up-and-Go (TUG) test was used to measure functional mobility. The Bruce treadmill test determined the participants’ submaximal endurance capacity. Data were analysed using mixed model repeated measures ANOVA and *P* < 0.05 was considered statistically significant.

**Results:**

Main treatment effects were found for muscle strength (*P* < 0.001) and functional mobility (*P* < 0.05). Upper and lower body strength generally showed a statistically significant improvement after every 4 weeks in RT (the increase after 16 weeks being 7.3 ± 4.9 kg and 86.6 ± 44.4 kg, respectively; *P* < 0.001) while TUG performance (−0.2 ± 0.4 s; *P* < 0.05) and submaximal endurance capacity (0.7 ± 0.9 min; *P* < 0.001) only improved after 16 weeks. Although muscle strength decreased after DET, it was still better than at baseline. No significant improvements in any performance variable were observed in CON directly after the intervention period (0–16 weeks) (*P* > 0.05).

**Conclusion:**

A 16-week RT programme has positive effects on both muscular and physical function in older adults, although the time course of these adaptations is different. While the gains in muscle strength and submaximal endurance capacity were not totally lost after DET, functional mobility was completely reversed. Older adults can be reassured that if the need arises to discontinue RT for a certain period they will still retain a large amount of their acquired muscle strength, as well as a degree of physical function such as submaximal endurance capacity. The association between leg strength and submaximal endurance capacity strengthens the notion that RT should be incorporated in training and rehabilitation programmes of ageing and frail older adults.

## Introduction

Muscular function plays an important role in the retention of adequate physical functioning with ageing [1, 2]. An individual’s muscle strength capacity also plays an essential role in maintaining an independent lifestyle [3, 4]. The preservation of a sense of independence subsequently results in a reduction in the burden on family, the public health sector and the national economy [5].

A 2012 report by Westcott highlights the benefits of resistance training (RT), which are of particular importance to the elderly population [1]. It was concluded that resistance exercise can reverse or delay age-related declines in physical and mental health. Another important benefit of RT, as stated in a review by Liu-Ambrose & Donaldson [6], is moderating the development of sarcopenia – a phenomenon known to increase fall risk and dependence among the elderly.

As stated in a 2009 ACSM Position Stand, untrained individuals experience the greatest gains in muscle strength when performing three exercise sessions per week [7]. Nakamura et al. [8] recommended three training sessions per week, consisting of a combination of RT, walking and recreational activities, to achieve improvements in functional fitness. Significant increases in 1RM values have been reported after 6 and 12 weeks of strength training, which was associated with a concomitant increase in functional capacity gains [9–11]. However, the majority of studies merely test muscle strength and physical function before and after the intervention period [9, 10, 12–14]. To our knowledge, only one study assessed muscle strength every 4 weeks during a 16-week training programme. However, the detraining (DET) period was very short (4 weeks), only lower body exercises were performed and no measure of functional mobility was included [15]. Thus, the manner in which changes in upper and lower body muscle strength and functional mobility are induced over the course of a resistance training intervention is still not known.

The degree to which the observed improvements in muscle strength and physical function can be maintained after the cessation of RT has not been thoroughly investigated. Researchers report significant losses in muscle strength after several weeks of inactivity following RT interventions, with DET periods ranging from 6 weeks to 18 months [10, 16, 17]. In some instances, however, the strength measured after DET was still significantly higher than at baseline [16, 17], while others report a complete loss of muscle strength gains at follow-up [10]. Geirsdottir et al. [16] found that TUG performance was maintained after a DET period of 6 to 18 months. In fact, participants performed functionally better at follow-up than before the start of the intervention.

It is difficult to draw inferences from existing longitudinal studies regarding the time course of changes in older adults’ muscular and physical function, as it has not been previously documented collectively. The primary aim of this study was therefore to assess the magnitude of changes in upper and lower body muscle strength, functional mobility and submaximal endurance capacity in older individuals during 16 weeks of RT, as well as a similar duration DET period. The secondary aim was to determine if there is a difference in the time course of the observed changes during training and DET.

## Methods

### Participants

Inactive men and women between 55 and 75 years who volunteered for this pre-post measures experimental-control research study underwent a screening procedure to identify those who met the inclusion criteria. They were screened for co-morbidities to minimize external influences on the training responses and possible risks to themselves. The co-morbidities were assessed by means of non-fasting cholesterol and glucose tests, anthropometry and cardiovascular measures, as well as a health questionnaire. Participants were included in the study if they had a body mass index (BMI) of less than 35 kg/m^2^ and had not been participating in at least 30 min of moderate intensity physical activity (64–76 % of maximal heart rate) on at least three days of the week for at least 3 months, as advised by the ACSM’s Guidelines for Exercise Testing and Prescription [18]. Participants were excluded if they had one or more signs/symptoms of, or diagnosed cardiovascular, pulmonary and/or metabolic diseases, if they experienced orthopaedic or musculoskeletal problems that could affect their exercise ability and if they achieved a Montreal Cognitive Assessment (MoCA) score of less than 26 out of 30. The study proposal was approved by the Ethics Committee of Stellenbosch University (HS891/2013).

Of the 61 subjects who were screened, a total of 46 met the inclusion criteria and were randomly assigned to either a resistance training (RT) group or a non-exercise control (CON) group by means of a randomised block design. All participants were informed of the purpose of the study and gave written consent to participate. Two participants dropped out of the RT group, while three did not want to participate because they were included in the CON group. Thus, 41 men and women (mean age 62.4 ± 5.3 years; BMI 26.3 ± 3.9 kg/m^2^) completed the intervention, with 22 participants in the RT group and 19 in the CON group.

### Testing protocol

Participants were assessed at six different time points throughout the intervention: at baseline (BL), every 4 weeks (week 4–12), at the end of the intervention period (week 16) and after a detraining (DET) period of 16 weeks. All participants were asked to maintain their current lifestyle and not make any changes to their level of physical activity and diet. The participants in the CON group were reminded of these conditions at each testing session.

Muscular strength, functional mobility and submaximal endurance capacity were measured as primary outcome variables. Participants were asked to void their bladders and to refrain from smoking, exercise and drinking diuretics like caffeine or alcohol for at least 4 h before the tests.

A resting ECG, waist-to-hip ratio, standing height, body mass and the MoCA were administered during the first visit as screening tests. During the second visit (BL-testing) the Timed-Up-and-Go (TUG) test was administered to assess functional mobility. The participant was instructed to sit on a standard chair. On the command “Go”, he/she stood up from the chair, walked three meters forward, turned and walked back to the chair. Timing started when the command was given and stopped when the subject was again sitting in the chair. Each participant performed three trials and the fastest time was noted as the final result.

The participant’s submaximal endurance capacity was assessed on the h/p/cosmos Saturn treadmill (Nussdorf-Traunstein, Germany) using the modified Bruce protocol. Heart rate was recorded with a Suunto memory belt (Suunto Oy 11/2007, Finland). The test started at an incline of 10° and a speed of 2.7 km/h. The incline and speed were increased incrementally every 3 min until the target heart rate (THR) of 75 % of the age-predicted maximal (220-age) was reached. The participant’s rating of perceived exertion (RPE) was recorded at the end of each stage and when the THR was reached. Participants then actively cooled down for 5 min at 2.7 km/h and no gradient.

Each participant also completed a familiarization session for the muscle strength tests to ensure proper technique and to avoid the Valsalva manoeuvre. The 10 repetition maximum (10RM) bench press and leg press tests were performed during the third session to determine the maximal upper and lower body muscle strength. These results were used to determine the initial intensity of the resistance exercises of the training programme. An initial light load was estimated, considering the subject’s RPE score following the warm-up set, which allowed the individual to complete ten repetitions comfortably. The load was then progressively increased until only 10 repetitions could be completed. The 10RM tests were repeated every 4 weeks to ensure that participants were exercising at the required intensity for the duration of the intervention period. Exercise sessions commenced from the fourth visit onwards.

### Training programme

The intervention was conducted over a period of 16 weeks and participants completed three 40-min sessions per week. Seven resistance exercises were performed using machines and free weights, alternating muscle groups (incline leg press, bench press, squat, latissimus dorsi pull-down, seated row, seated hamstring curls and a seated shoulder press). Three sets of 10 repetitions were performed for each exercise with a rest period of 30 s between each set and 90 s between each exercise. The first set was performed at 50 % of the individual’s 10RM, the second set at 75 % and the third set at 100 % of the 10RM. After 8 weeks the load for each set was increased to 75, 85 and 100 % of the individual’s 10RM, respectively. The RPE scale was used to monitor the participants’ subjective feeling regarding the intensity of the exercise and this value was recorded after completion of each set. An RPE rating of “moderate” was desired after the first set, followed by “somewhat hard” after the second and “hard” after the third set. Passive stretching concluded each session. The participant’s blood pressure was monitored before and after each exercise session as a safety precaution.

### Follow-up testing

The DET tests were completed 16 weeks after the post-intervention testing. A total of 19 participants in the RT group and 16 participants in the CON group participated in the follow-up testing. The TUG test, modified Bruce protocol and 10RM bench press and leg press tests were performed.

## Statistical analysis

Statistical analysis was performed using STATISTICA 12. Mixed model repeated measures ANOVA was used to analyse the data. The participants were entered in the model as random effects and treatment and time as fixed effects. Fisher least significant difference (LSD) post hoc testing was used. A *P* value of < 0.05 was considered statistically significant. Of all the types of post hoc testing one can do, Fisher LSD provide the least protection for multiple testing but it was deemed most appropriate as the post hoc tables contained many comparisons due to the six time points that were included in the design. Other post hoc methods could render too many p-values non-significant in such cases, which in turn creates power issues. Pearson product–moment correlation coefficients and 95 % confidence intervals were calculated for the relationships between the change in upper and lower body strength, functional mobility and submaximal endurance capacity [19]. The smallest practically significant correlation was set at ± 0.1. All values are reported as means ± SD.

## Results

There were no statistically significant differences in the baseline (BL) characteristics of the resistance training (RT) and control (CON) groups (*P* > 0.05) (Table 1).

**Table 1:** Baseline characteristics of the participants (mean ± SD)

Variable	RT group	CON group	Total
n	22	19	41
Age (years)	62.4 ± 5.1	62.5 ± 5.6	62.4 ± 5.3
Height (cm)	167.8 ± 7.8	168.7 ± 7.9	168.2 ± 7.9
Body mass (kg)	73.3 ± 15.5	76.8 ± 13.7	74.9 ± 14.8
BMI (kg · mˉ^2^)	25.8 ± 4.0	26.9 ± 3.7	26.3 ± 3.9
10RM leg press	70.5 ± 39.4	81.3 ± 41.8	75.5 ± 40.9
10RM bench press	22.7 ± 14.3	21.2 ± 9.0	22 ± 12.2

No statistically significant differences in the physical characteristics of the RT and CON groups at BL (*P* > 0.05)

*RT* resistance training, *CON* control, *BL* baseline, *BMI* body mass index, *RM* repetition maximum

**Fig. 1:**
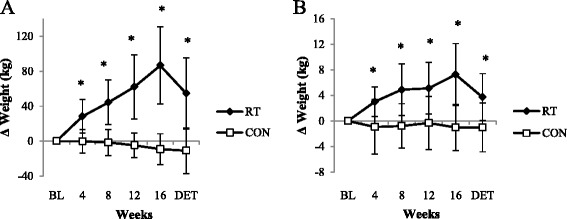
Relative changes in muscle strength. Changes in lower body strength (**a**) and upper body strength (**b**) from BL in RT and CON during the 16-week intervention and after the DET period. *Statistically significant between-group differences (*P* < 0.001)

Following the 16 weeks of training, the RT group showed a statistically significant increase in lower body strength (86.6 ± 44.3 kg; *P* < 0.001) (Table 2). In fact, the change in muscle strength was statistically significant after each month of training (*P* < 0.05), as depicted in Fig. 1a, while no improvement was observed in the CON group (−9.2 ± 17.7 kg; *P* > 0.05). A significant Time x Group interaction was found for lower body strength (*P* < 0.001.)

**Table 2:** Within-group comparisons for muscle strength, functional mobility and submaximal endurance capacity (mean ± SD)

	Weeks of training					
Variable	BL	4	8	12	16	DET
Lower body strength (kg)						
RT	70.5 ± 39.4	98.9 ± 44.5^*^	114.8 ± 50.5^*^	132.5 ± 58.2^*^	157.1 ± 69.1^*^	123.7 ± 56.4^*^
CON	81.3 ± 41.8	81.1 ± 42.8	79.7 ± 42.6	76.6 ± 39.0	72.1 ± 36.1	72.5 ± 33.2^**^
Interaction effect: *P* < 0.001						
Upper body strength (kg)						
RT	22.7 ± 14.4	25.7 ± 15.4^*^	27.6 ± 15.1^*^	27.8 ± 15.0^*^	30.0 ± 16.4^*^	25.2 ± 10.8^*^
CON	21.2 ± 9.0	20.3 ± 8.7	20.4 ± 8.6	20.9 ± 9.8	20.2 ± 8.6	21.5 ± 9.2
Interaction effect: *P* < 0.001						
TUG (s)						
RT	5.4 ± 0.9	5.3 ± 0.8	5.3 ± 0.7	5.5 ± 0.8	5.1 ± 0.8^**^	5.4 ± 0.8
CON	5.5 ± 1.1	5.6 ± 0.9	5.7 ± 1.0	5.7 ± 0.9	5.7 ± 0.8^**^	5.6 ± 0.8^**^
Interaction effect: *P* < 0.05						
Time to THR [Bruce test (min)]						
RT	5.5 ± 1.6				6.2 ± 1.4^*^	6.4 ± 2.0^*^
CON	5.8 ± 1.6				5.8 ± 1.6	6.4 ± 1.9^**^
Interaction effect: *P* > 0.05						

*BL* baseline, *DET* detraining, *RT* resistance training, *CON* control, *TUG* timed-up-and-go, *THR* target heart rate

^*^Significantly different from BL (*P* < 0.001)

^**^ *S*ignificantly different from BL (*P* < 0.05)

Although the RT group lost a significant amount of lower body strength after the detraining (DET) period (34.0 ± 23.5 kg; *P* < 0.001) (Table 2), this value was still significantly higher than their BL values (52.6 ± 23.5 kg; *P* < 0.001), as well as the CON group’s DET value (*P* < 0.001) (Fig. 1a).

The RT group improved their upper body strength significantly after 16 weeks (7.3 ± 4.9 kg; *P* < 0.001), while no change was observed in the CON group (−1.0 ± 3.6 kg; *P* > 0.05) (Table 2). With the exception of the second to third month, the increase in muscle strength was significant after each month of training (*P* < 0.05). A significant Time x Group interaction was found for upper body strength (*P* < 0.001).

After DET, the RT group’s upper body strength decreased significantly (3.8 ± 3.4 kg; *P* < 0.001), but this value was still significantly higher compared to their BL values (3.5 ± 3.4 kg; *P* < 0.001) (Table 2). The between-group difference from BL to DET was also statistically significant (increase of 3.5 ± 3.4 kg in RT vs decrease of 1.0 ± 3.3 kg in CON; *P* < 0.001).

**Fig. 2:**
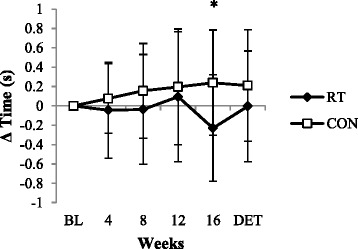
Relative changes in functional mobility. Changes in TUG performance from BL in RT and CON during the 16-week intervention and after the DET period. *Statistically significant between-group difference at post-test (*P* = 0.01)

The RT group’s Timed-Up-and-Go (TUG) performance improved significantly after the intervention period (−0.2 ± 0.4 s; *P* < 0.05), while the CON group experienced a significant decline in TUG performance (0.3 ± 0.4 s; *P* < 0.05) (Table 2). However, after DET the gain in functional mobility in the RT group was completely lost and values were back to BL (*P* > 0.05) (Fig. 2). A significant Time x Group interaction was found for functional mobility (*P* < 0.05).

**Fig. 3:**
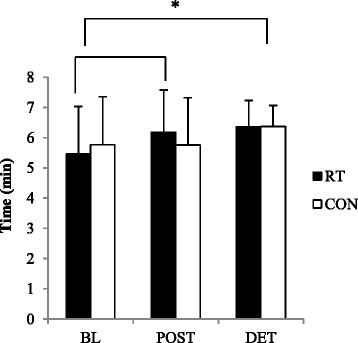
Time to reach target heart rate (THR) during the Bruce treadmill test. No differences were observed between the groups (*P* > 0.05). *RT improved their time to reach THR following the intervention period (*P* < 0.001). RT and CON showed an increased time to reach THR after DET compared to BL (*P* < 0.05)

There was a statistically significant improvement in submaximal endurance capacity in the RT group after the intervention period (0.7 ± 0.9 min; *P* < 0.001), with a further improvement of 0.2 ± 0.9 min after the DET period (*P* < 0.001 from BL) (Table 2). Although the CON group had no change in their submaximal endurance capacity after the intervention period (*P* > 0.05), they performed significantly better after the DET period than at BL (0.6 ± 0.9 min; *P* < 0.05) (Fig. 3). The Time x Group interaction for submaximal endurance capacity did not reach significance (*P* > 0.05).

A moderate positive relationship was found between increases in leg strength and the improvement in submaximal endurance capacity (r = 0.54, CI: 0.27–0.72), while the relationship between increases in arm strength and the improved submaximal endurance capacity was not significant (r = 0.19, CI: −0.13–0.47). It is unlikely that gains in leg or arm strength have a meaningful practical influence on the enhancement in functional mobility (TUG) (r = −0.24, CI: −0.51–0.07 and r = −0.09, CI: −0.39–0.22, respectively).

## Discussion

Three main findings of this study were that (a) the time course for improvements in upper and lower body muscle strength, functional mobility and submaximal endurance capacity over 16 weeks was different; (b) gains in muscle strength and submaximal endurance capacity were not completely lost after 16 weeks of detraining (DET) and (c) leg muscle strength is an important correlate to submaximal endurance capacity in older individuals.

It was found that both arm and leg muscle strength improved significantly (39 ± 27 % and 167 ± 125 %, respectively) over the course of 16 weeks in older individuals who had no previous experience in resistance training (RT). The observed changes were already significant after 4 weeks; thereafter more gains were achieved every 4 weeks until the completion of the programme. The increase in the participants’ leg strength was significant after each month of training, suggesting that the progression in training loads for the leg exercises were well structured for the participants. Furthermore, seeing a steady monthly improvement in muscle strength will very likely be a strong motivating factor for older adults. The results support previous findings that noteworthy muscle strength increases can be achieved within 12 sessions in ageing individuals [15, 20], irrespective of training frequency per week. Pinto et al. [11] reported an improvement in the leg strength of elderly women after completing 12 lower body strength training sessions, while Lovell et al. [15] observed a similar improvement in older men. The latter study also reported a significant improvement in leg strength after each month of training over a 16-week training period. The present study adds to the existing literature by showing that 12 RT sessions result in a marked increase in upper body muscle strength and that after 48 sessions there was still no indication of a plateau in the improvements of both upper and lower body strength.

Modest, but statistically significant improvements in functional mobility [Timed-Up-and-Go (TUG) test] and submaximal endurance capacity during the Bruce test were observed only after 16 weeks of RT (3 ± 10 % vs 19 ± 29 %, respectively). These results are in contrast with previous findings where significant changes in physical function were evident after a shorter time period. Pinto et al. [11] found significant increases in physical function and muscle strength after 6 weeks of lower body strength training in elderly women, while others reported improved physical function after 12 weeks RT [9, 10]. The fact that a significant improvement in functional mobility in the current study was only observed at the end of the intervention may possibly be attributed to the fact that the RT programme did not focus exclusively on the leg muscles, but also included arm exercises. Furthermore, studies also vary in terms of training intensities, with this study employing higher exercise intensities than most others. In a meta-analysis Steib et al. [21] concluded that although higher training intensities (> 60 % 1RM) lead to greater gains in muscle strength, it is not necessarily more advantageous for improvements in physical function in older adults compared to low and moderate intensity RT. It should also be noted that only Pinto et al. [11] included a control group in their studies. Without a control group one cannot be certain that enhanced performances can be attributed to the training programme alone, as one cannot exclude the possibility of the Hawthorne effect, the learning effect, or normal day-to-day variation as reasons for the findings.

The participants’ improvement in submaximal endurance capacity in response to RT is in agreement with previous reports. An increased submaximal endurance capacity after RT could be the result of peripheral adaptations in the trained muscles, which have been reported in other RT studies. Lovell et al. [15] found an increase in arterial-venous oxygen difference after 16 weeks of strength training, while cardiac output remained unchanged. Researchers suggested that this increased ability of the muscles to utilize oxygen is the result of increases in capillary density and mitochondria in the trained muscles [15, 22]. It has also been proposed that resistance training results in the recruitment of less motor units by the working muscle, consequently prolonging the onset of total muscle fiber fatigue [14, 22].

Even though there was a decrease in the participants’ muscle strength at follow-up, their level of strength was still significantly higher than the values obtained at the pre-test. This finding is also in line with previous research. Despite a significant decrease in muscle strength after 20 weeks of DET following an 18-week progressive RT intervention, Harris et al. [17] reported that their participants’ muscle strength was still significantly higher compared to baseline values. The same trend was described by Geirsdottir et al. [16], where participants completed DET tests over a period of 6 to 18 months following a 12-week RT programme. These findings reflect the long lasting effects of well-designed progressive RT programmes and suggest that even if individuals cannot train for a period of time, all is not lost.

Functional mobility returned to pre-training values after 16 weeks of DET, despite the significant retention of leg strength. This finding is in agreement with the results of Correa et al. [10], but in contrast to the findings of Geirsdottir et al. [16]. Both studies investigated the effects of 12 weeks of RT, which was followed by DET periods of 12 months and longer. The inconsistencies in these findings could be a function of the differences in the frequency, duration and intensity of the interventions, as well as the follow-up periods. Furthermore, it should be noted that different functional mobility tests are used in the various studies and it is questionable whether the outcomes for these different tests (i.e., TUG, 30s sit-to-stand, stair climbing etc.) are comparable.

Table 2 shows that there were significant improvements in submaximal endurance capacity in both groups after the follow-up period. However, upon closer inspection of the data it was evident that only two participants in each group showed a pronounced improvement from post-test to DET [19.5 and 28 % in RT group and 67.2 and 24.8 % in control (CON) group], consequently affecting the group’s overall results. When these outliers are omitted from the data set, it shows that both groups did indeed perform better after DET, however, the improvements in performance were not statistically significant.

Our findings suggest that enhanced leg muscle strength is a better determinant of submaximal endurance capacity, than of functional mobility as assessed by the TUG test. This is probably because performance in the TUG test is more dependent on coordination, balance and reaction time, which are not necessarily enhanced by RT.

This is the first study to show that gains in functional mobility and muscle strength do not happen simultaneously. Whereas upper and lower body strength was significantly enhanced after 4 weeks, the improvement in functional mobility was only evident after 16 weeks. Longer term intervention studies (> 4 weeks) only reported pre- and post-training results and therefore it is unclear if this finding is unusual. It is not clear if this pattern is a function of our specific training programme, whether it is due to differences in the time course of physiological adaptations (i.e., peripheral and central adaptations), whether it is a finding limited to our population (i.e., low strength levels, but higher levels of functional mobility at the beginning of the study), or if it is a function of the sensitivity of the selected physical tests to change. According to the relative norms for upper and lower limb strength [18], the participants’ overall muscle strength was below average for men and women in both the 50–59 and greater than 60 years age categories prior to the intervention, while their TUG results were above average [23]. Therefore, the participants in this study had greater capacity to improve their muscular function than their functional mobility. Future studies should determine if participants with lower levels of functional mobility show similar patterns of change over the course of a physical intervention programme compared to the current study.

## Conclusion

The findings of the present study demonstrate that a 16-week RT intervention in ageing individuals is associated with significant improvements in upper and lower body muscle strength, as well as physical function, however, these changes do not come about concurrently. Older individuals can be reassured that if the need arises to discontinue RT for a certain period they will still retain a large amount of their acquired muscle strength, as well as a degree of physical function such as submaximal endurance capacity. The association between leg strength and submaximal endurance capacity strengthen the notion that RT should be incorporated in training and rehabilitation programmes of ageing and frail older adults.

## Study limitations

Differences in the baseline levels of the experimental group’s muscle strength and functional capacity could be considered a limiting factor, due to the possible ceiling effect on the TUG test. Furthermore, the TUG test might not have adequate sensitivity to detect changes in functional mobility in a sample consisting of highly functional older adults. The omission of a test for functional mobility as an inclusion criterion adds another limitation to this study and should be considered in future investigations.

A specific measure of the participants’ upper body physical function was also not included; however, the TUG test was included as it is universally recognised as a good determinant of overall functional mobility.
